# Model‐based data analysis of individual human postprandial plasma bile acid responses indicates a major role for the gallbladder and intestine

**DOI:** 10.14814/phy2.14358

**Published:** 2020-03-13

**Authors:** Emma C. E. Meessen, Fianne L. P. Sips, Hannah M. Eggink, Martijn Koehorst, Johannes A. Romijn, Albert K. Groen, Natal A. W. van Riel, Maarten R. Soeters

**Affiliations:** ^1^ Department of Endocrinology and Metabolism Amsterdam University Medical Centers, Academic Medical Center (AMC) Amsterdam The Netherlands; ^2^ Department of Biomedical Engineering Eindhoven University of Technology Eindhoven The Netherlands; ^3^ Department of Laboratory Medicine Center for Liver Digestive and Metabolic Diseases University Medical Center Groningen Groningen The Netherlands; ^4^ Department of Internal Medicine Amsterdam University Medical Centers, Academic Medical Center (AMC) The Netherlands; ^5^ Department of Vascular Medicine Amsterdam University Medical Centers Amsterdam, Academic Medical Center (AMC) The Netherlands

**Keywords:** interindividual variability, intraindividual variability, mathematical modeling, mixed meal test, postprandial bile acid metabolism

## Abstract

**Background:**

Bile acids are multifaceted metabolic compounds that signal to cholesterol, glucose, and lipid homeostasis via receptors like the Farnesoid X Receptor (FXR) and transmembrane Takeda G protein‐coupled receptor 5 (TGR5). The postprandial increase in plasma bile acid concentrations is therefore a potential metabolic signal. However, this postprandial response has a high *interindividual* variability. Such variability may affect bile acid receptor activation.

**Methods:**

In this study, we analyzed the *inter*‐ and *intraindividual* variability of fasting and postprandial bile acid concentrations during three identical meals on separate days in eight healthy lean male subjects using a statistical and mathematical approach.

**Main findings:**

The postprandial bile acid responses exhibited large *interindividual* and *intraindividual* variability. The individual mathematical models, which represent the enterohepatic circulation of bile acids in each subject, suggest that *interindividual* variability results from quantitative and qualitative differences of distal active uptake, colon transit, and microbial bile acid transformation. Conversely, *intraindividual* variations in gallbladder kinetics can explain *intraindividual* differences in the postprandial responses.

**Conclusions:**

We conclude that there is considerable *inter*‐ and *intraindividual* variation in postprandial plasma bile acid levels. The presented personalized approach is a promising tool to identify unique characteristics of underlying physiological processes and can be applied to investigate bile acid metabolism in pathophysiological conditions.

## Graphical abstract

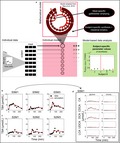

**Abbreviations**
AUC: area under the curve
BMI: body mass index
CA: cholic acid
CDCA: chenodeoxycholic acid
CV: coefficient of variation
DCA: deoxycholic acid
FXR: farnesoid X receptor
GLP‐1: glucagon‐like peptide‐1
iAUC: incremental area under the curve
LCA: lithocholic acid
MMT: mixed meal test
NASH: nonalcoholic steatohepatitis
T2DM: type 2 diabetes mellitus
TGR5: takeda G protein‐coupled receptor 5
UDCA: ursodeoxycholic acid

**Key points summary**

‐ Postprandial bile acids show *inter*‐ and *intraindividual* variation to mixed meal testing in postprandial plasma bile acid levels in healthy lean men.
‐ Model‐based analysis suggests quantitative and qualitative differences of distal active uptake, colon transit, and microbial bile acid transformation contribute to *interindividual *variability.
‐ *Intraindividual* variations in gallbladder kinetics can explain *intraindividual* differences in the postprandial responses.
‐ Personalized mathematical modeling of postprandial plasma bile acid responses allows the identification of qualitative and quantitative characteristics of individual bile acid metabolism.

## 1 INTRODUCTION

In the last two decades bile acids have gained attention in metabolic research because of their proposed postprandial signaling via the intranuclear Farnesoid X Receptor (FXR) and transmembrane Takeda G protein‐coupled receptor 5 (TGR5) (Kuipers, Bloks, & Groen, 2014; van Nierop et al., 2017). Via these receptors, bile acids may affect glucose, lipid, and energy metabolism both in health and diseases such as nonalcoholic steatohepatitis (NASH) (Zhu, Liu, Zhang, & Guo, 2016) and Type 2 diabetes mellitus (T2DM) (van Nierop et al., 2017).

The primary bile acids, cholic acid (CA), and chenodeoxycholic acid (CDCA), are synthesized from cholesterol in hepatocytes, secreted into bile after glycine or taurine conjugation, and subsequently stored in the gallbladder. In response to nutrient ingestion, bile is released into the duodenum via gallbladder emptying and facilitates the digestion of dietary fat and fat‐soluble vitamins (Lefebvre, Cariou, Lien, Kuipers, & Staels, 2009). Up to 95% of the bile acids are reabsorbed from the small intestine, mostly via the apical sodium‐dependent bile acid transporter (ASBT) (Hofmann & Hagey, 2008). The 5% that escapes re‐uptake can be converted into secondary bile acids (i.e., deoxycholic acid [DCA], lithocholic acid [LCA], and ursodeoxycholic acid [UDCA]) via deconjugation, dehydroxylation, and further transformation by gut microbiota and are found primarily in the colon (Winston & Theriot, 2019). These newly created secondary bile acids are either excreted in the feces or passively absorbed in the colon. The liver extracts ~95% of the bile acids from the portal vein, and secretes these into the bile, completing the enterohepatic cycle (Eggink et al., 2017; Sips et al., 2018). A small proportion of bile acids escapes this enterohepatic cycle and reaches the systemic circulation in concentrations that are less than 20% of what is found in the portal vein (Eggink et al., 2017; Sips et al., 2018).

The postprandial increase in bile acid concentrations as a result of gallbladder emptying is a potential metabolic signal within the enterohepatic cycle (Kuipers et al., 2014). Plasma bile acid concentrations consistently increase after a mixed meal test (MMT), but the timing, shape, and bile acid composition of the postprandial curve shows high variability between subjects (*interindividual* variability) (Al‐Khaifi et al., 2018; Eggink et al., 2017; Gälman, Angelin, & Rudling, 2005; LaRusso, Korman, Hoffman, & Hofmann, 1974; Sonne et al., 2016; Steiner et al., 2011). It is unclear what underlies postprandial bile acid variability, as in silico analysis has demonstrated various factors influence postprandial response of bile acids (Sips et al., 2018).

In this study, we characterized the variability of the postprandial bile acid responses and investigated this variability via a personalized modeling approach. To this end, we first assessed the *inter*‐ and *intraindividual* variability of fasting and postprandial bile acid concentrations during three consecutive identical meals on separate days in eight healthy lean men. Subsequently, this detailed mapping of the *intraindividual* variability allowed us to develop an individual mathematical modeling procedure for in depth data analysis, based on the population‐level enterohepatic circulation model we have developed previously (Sips et al., 2018). Individual model‐based analysis was employed to investigate the sources of the postprandial variation. These models suggest that *intraindividual* variation in gallbladder emptying kinetics is sufficient to explain *intraindividual* differences in postprandial response. *Interindividual* variability in the personal models stems from quantitative and qualitative differences of distal active uptake and colon transit parameters. This personalized approach may identify unique characteristics of underlying physiological processes and could be applied to investigate bile acid metabolism in pathophysiological conditions.

## 2 MATERIAL AND METHODS

### 2.1 Subjects

We included eight healthy male subjects who all completed the study. Subjects with previous biliary surgery or current liver, biliary or gastrointestinal disease were excluded. Other exclusion criteria were substance use (nicotine, drugs, or alcohol >3 units/day), medication or herbal supplement use and exercise (defined as >1 hr per day) 3 days prior to the study days. Oral and written informed consent were obtained from all subjects before the start of the study in accordance with the principles of the Declaration of Helsinki (2013). The study was approved by the Medical Ethics Committee of the Academic Medical Center (AMC), Amsterdam, The Netherlands.

### 2.2 Experimental procedures

The study was performed in January 2017 at the Experimental and Clinical Research Unit (ECRU) of the Amsterdam UMC, location AMC. Subjects underwent three MMTs on 3 separate days within a period of 2 weeks. Nutridrink Compact (Nutricia, Zoetermeer, The Netherlands) was used as a standardized liquid mixed meal, containing 49% carbohydrates, 35% fat, and 16% protein. The liquid mixed meal consisted of 25% of the individual estimated daily energy expenditure, calculated using the Harris‒Benedict equation (Harris & Benedict, 1918) and then multiplied by 1.3 to correct for activity. After an overnight fast, subjects attended the ECRU by car or public transport at 08:00 hr and a cannula was placed into the antecubital vein for blood collection. The MMT started at 09:00 hr when the subjects consumed the liquid mixed meal.

### 2.3 Data collection and analytical procedures

For bile acid analyses, venous blood samples were drawn just before and 15, 30, 45, 60, 75, 90, 120, 150, 180, and 240 min after the ingestion of the liquid mixed meal (t = 0). Blood was collected into EDTA tubes and immediately kept on ice, centrifuged for 15 min 3000*g*, 4ºC) and then stored on −20°C until analyses. Bile acid concentrations were determined using a LC/MS/MS method (Eggink et al., 2017).

### 2.4 Statistical analyses

Total bile acid concentrations were calculated as the sum of the unconjugated‐ and conjugated (glycine‐ and taurine) forms of the primary bile acids CA, CDCA and the secondary bile acids DCA, LCA and UDCA. The area under the curve (AUC) and incremental AUC (iAUC) (using baseline subtracted concentrations) were calculated using the trapezoidal rule (Louton, Kuhnz, Dibbelt, & Knuppen, 1994). We did not include the taurine‐conjugated forms in our statistical analysis since they were hardly detectable in plasma.

A formal power analysis was not performed since this study was designed to observe the *inter*‐ and *intraindividual* variability of the postprandial response, and not to assess a difference from for example an intervention.

We first assessed comparisons of all the postprandial responses between the three meals (*N* = 3 meals*8 subjects = 24 meals) with the two‐way repeated measures ANOVA. Second, we compared the AUC and iAUC with the one‐way repeated measures ANOVA when iAUCs were normally distributed. When data were not normally distributed the Friedman test was used. Bonferroni testing was performed as post hoc analysis for the ANOVAs whereas the Dunn's test was applied for the Friedman test.

**Figure 1:**
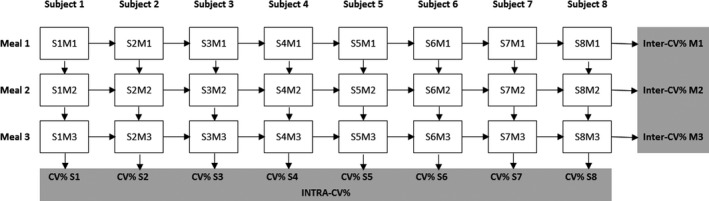
Schematic overview of the calculated inter‐ and intracoefficients of variance to the three identical liquid mixed meal tests in eight healthy lean men. The individual intra‐CVs are calculated from the standard deviation divided by the mean of three meals of one subject. The intra‐CV is the mean and 95% confidence interval of all the individual intra‐CVs. The three inter‐CVs are calculated from the standard deviation divided by the mean of the measured parameter of all subjects on the specific meal day. All CVs are multiplied by 100 to create a percentage. CV, coefficient of variance; M, meal; S, subject

To assess variability, we used the coefficient of variance (CV) of the AUC’s and iAUC’s, individual time points and peak concentrations. The MMT variability (CV%) was assessed from the means of the three meals and calculated from the standard deviation divided by the mean and then multiplied by 100. Furthermore, we calculated *interindividual* variability (inter‐CV%) and *intraindividual* variability (intra‐CV%). Figure 1 gives a schematic overview of the calculated inter‐ and intra‐CVs. The CV’s can be 0% or greater and we considered a CV >20% as high variability (Krug et al., 2012) since, to our knowledge, no previous cut‐off values were published. The unconjugated bile acids were essentially unchanged in the postprandial state and therefore we could not calculate the inter‐ and intra‐CV of the iAUC.

Data are presented as mean and standard deviation (*SD*) when normally distributed (tested with the Shapiro‒Wilk test) or otherwise the median and interquartile range [IQR] are given. Intra‐CVs are presented as mean and confidence interval (CI) 95%. Statistical analysis was performed with IBM SPSS Statistics 25 (IBM) and Graphpad Prism 7.02 (GraphPad Software). Graphs were designed using Graphpad Prism 7.02. We considered a p‐value below 0.05 as statistically significant.

### 2.5 Mathematical modeling

A subject‐specific mathematical model was created for each subject based on the model previously published (Sips et al., 2018). The model was adapted for use in individual fashion in several ways: (a) Several model parameters were removed or fixed based on the available data and previous analysis (Appendix 1), (b) Postprandial gallbladder emptying, conversely, was expanded for liquid meals, (c) The resulting free model parameters were finally divided into 22 subject‐specific parameters (allowed to vary between subjects) and 4 meal‐specific parameters (Allowed to vary not only between subjects, but also between a single subject's three distinct meals). Meal‐specific parameters were incorporated to reflect the large *intraindividual* variability found in postprandial responses—see Results. All meal‐dependent parameters control gallbladder emptying, to allow large intrasubject variability in stomach (Yokrattanasak et al., 2016) and gallbladder emptying (Schiedermaier, Neubrand, Hansen, & Sauerbruch, 1997). The values of the free parameters per subject‐specific model were determined by minimizing a cost function summing the absolute difference between simulated individual bile acid species plasma concentrations and corresponding measured concentrations over all time points for all three meals simultaneously.

### 2.6 Identifiability analysis

Parameter identifiability was analyzed to quantify uncertainty (Vanlier, Tiemann, Hilbers, & van Riel, 2013). Hereto, each subject‐specific optimization was performed 25 times. In addition to the optimal parameter set, parameter sets resulting in less than 25% higher cost function were also selected as part of a representative set of solutions. A measure of parameter identifiability was then defined by calculating the coefficients of variance (CV_par)_ within this set of parameter values. Well‐identifiable parameters were assumed to have lower than 20% variance, whereas parameters with more than 50% variance were considered nonidentifiable.

### 2.7 Sensitivity analysis

To determine which parameters and processes have the most influence on the postprandial profiles, we performed local sensitivity analyses with the optimal parameter set for each subject‐specific model. Sensitivity was calculated based on the total bile acid concentrations (Appendix 1). To determine sensitivity, parameters were changed by 0.01%. Sensitive parameters were then ranked to obtain a qualitative overview of the most influential parameters.

## 3 RESULTS

### 3.1 Subjects

**Figure 2:**
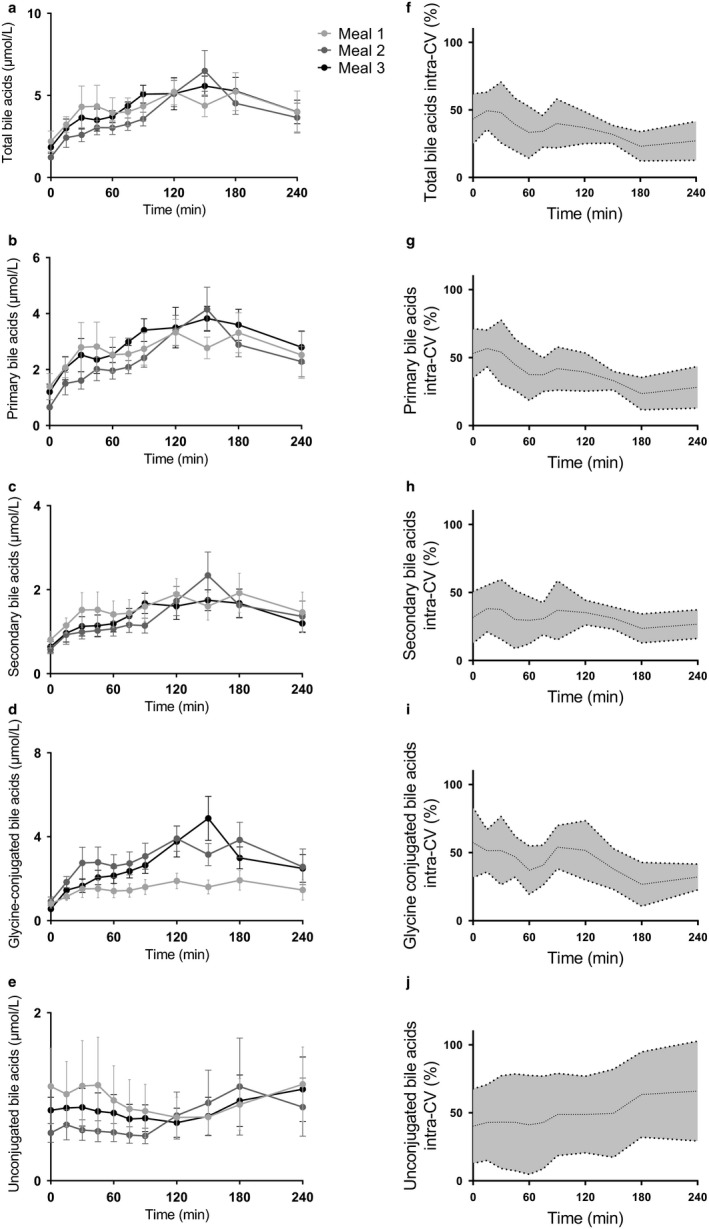
Postprandial bile acids responses and intracoefficicients of variance (intra‐CV) to the three identical liquid mixed meal tests in healthy lean men. In a crossover design, eight healthy lean men underwent three identical liquid mixed meal tests in a period of 2 weeks at T = 0 after an overnight fast. On the left panel, postprandial excursions of (a) total bile acids, (b) primary bile acids, (c) secondary bile acids, (d) glycine conjugated bile acids, and (e) unconjugated bile acids are shown. Each color represents a different meal and data are presented as mean and *SEM*. On the right panel, the intra‐CVs of (f) total bile acids, (g) primary bile acids, (h) secondary bile acids, (i) glycine‐conjugated bile acids, and (j) unconjugated bile acids are displayed. Data are presented as mean and 95% confidence interval

We included eight healthy lean young male subjects (Body Mass Index (BMI) 22.7 ± 1.3 kg/m^2^, age 23.5 ± 3.3 years; fasting glucose 4.4 ± 0.3 mmol/L; fasting insulin 43.9 ± 36.1 pmol/L). The individual fasted and postprandial bile acid concentrations showed considerable *inter‐* and *intraindividual* variation as depicted in Figure 2, Figure S1, Tables S1 and S2. An overview of *inter*‐ and *intraindividual* variability of the AUC’s and iAUC’s is shown in Table 1.

**Table 1:** The postprandial bile acid responses and its variability to three identical liquid mixed meal tests in eight healthy lean men

	Meal 1	Meal 2	Meal 3	*p*‐value	CV%	Inter‐CV%			Intra‐CV%
						Meal 1	Meal 2	Meal 3	
Total bile acids									
AUC	927 [420]	1,181 [616]	1,142 [443]	NS	5.8	53.1	35.0	22.6	20.5 [11.6 –29.4]
iAUC	763 [368]	999.5 [495.3]	936 [295]	NS	5.7	50.3	34.6	21.8	22.5 [15.0 – 29.9]
Primary bile acids									
AUC	575 [128]	678 [335]	790 [327]	NS	7.4	53.7	34.4	33.3	22.1 [12.9 – 31.3]
iAUC	446 [166]	487 [241]	565 [329]	NS	11.7	49.7	39.3	31.4	25.6 [17.2 – 34.0]
Secondary bile acids									
AUC	383 ± 224	344 ± 172	341 ± 127	NS	5.6	58.50	49.8	37.3	21.1 [12.5 –29.7]
iAUC	189 ± 126	206 ± 105	189 ± 93	NS	5.2	66.7	51.1	49.5	41.9 [23.6 – 60.2]
Glycine‐conjugated bile acids									
AUC	633[338]	686 [490]	825 [473]	NS	6.0	47.7	39.4	34.3	20.7 [12.4 – 29.1]
AUC	489 [346]	539 [454]	585 [391]	NS	8.2	43.7	43.0	37.4	23.6 [14.7 – 32.5]
Unconjugated bile acids									
AUC	152 [165]	154 [135]	135 [213]	NS	8.9	103.5	91.7	73.8	45.6 [18.0 – 73.2]
iAUC	0.3 [100]	8.6 [76.0]	6.6 [96]	NS	26.8	X	X	X	X

Notes

Data are presented as mean ± standard deviation when normally distributed or otherwise the median [interquartile range] is used. The inter‐ and intra‐CV are not displayed for the iAUC of the unconjugated bile acids since the iAUC was negative in our study.

Abbreviations: CV: coefficient of variance; iAUC: incremental area under the curve.

### 3.2 Total bile acid concentrations

Postprandial bile acid curves did not differ significantly between the three meals (Figure S1a, two‐way repeated measures ANOVA *p* > .05). The AUC’s and iAUC’s of the three meals were not different (Figure 2a, Table 1) and the mixed meal test variability of the AUC and iAUC was low (Table 1). However, *inter*‐ and *intraindividual* variability of the AUCs and iAUCs was high and differed between study days (Table 1). The fasted and postprandial intra‐CV for each time point was high and decreased over time from +15 min to 240 min after ingestion of the meal (Figure 2f, Table S1). Moreover, the intra‐CV of the peak concentrations was also high (Table S2).

### 3.3 Primary and secondary bile acid concentrations

The postprandial concentrations of primary and secondary bile acids did not differ significantly between the three meals (Figure S1b,c, two‐way repeated measures ANOVA *p* > .05). Furthermore, the AUCs and iAUCs for the three meals were not different (Figure 2b,c, table 1). The CVs of the primary and secondary bile acids AUC and iAUC were low (Table 1), corresponding to the CV of the total bile acid concentrations. The *inter‐* and *intraindividual* variability of the primary and secondary bile acids was high for the AUCs and iAUCs (Table 1). Remarkably, the *intraindividual* variability of the secondary bile acids’ iAUC is much higher (intra‐CV = 41.9%). Again, the *intraindividual* variability of the postprandial response of both the primary and secondary bile acids was high and showed a decrease in *intraindividual* variability similar as the total bile acid concentrations (Figure 2g,h, Table S1). The intra‐CV of the complete (all individual time points) postprandial secondary bile acids response was lower compared to the intra‐CV of the primary bile acids (Table S1, two‐way repeated measures ANOVA *p* < .01). The intra‐CV in peak concentrations of the primary and secondary bile acids was high (Table S2).

### 3.4 Glycine‐conjugated and unconjugated bile acid concentrations

The postprandial responses of glycine‐conjugated and unconjugated bile acids did not differ between the three meals (Figure S1d,e, two‐way repeated measures ANOVA *p* > .05). The AUCs and iAUCs of the three meals were not different for the glycine‐conjugated and unconjugated bile acids (Figure 2d,e, Table 1). The CV of the AUC between the three meals was low for the glycine‐conjugated and unconjugated bile acids (Table 1) Surprisingly, the CV for the iAUC of the unconjugated bile acids was 26.8% whereas all the other bile acids had a CV below <10% (Table 1). The inter‐ and intra‐CV of the glycine‐conjugated bile acids during the separate meals was high and differed between meals (Table 1). Interestingly, the postprandial response of the glycine‐conjugated showed a decrease in *intraindividual* variability whereas the unconjugated bile acids showed a postprandial increase in variability (Figure 2i,j, Table S1), however, this was not significant (two‐way repeated measures ANOVA *p* > .05) The *intraindividual* variability in peak concentrations of the glycine‐conjugated and unconjugated bile acids was high, especially for the unconjugated bile acids (Table S2). Therefore, the variability of the postprandial unconjugated bile acids response follows a different pattern compared to the other described bile acids (Figure 2f,j). The altered pattern of the unconjugated bile acids support the previous published asynchronous variations of unconjugated and conjugated bile acids (Al‐Khaifi et al., 2018).

### 3.5 Mathematical modeling

**Figure 3:**
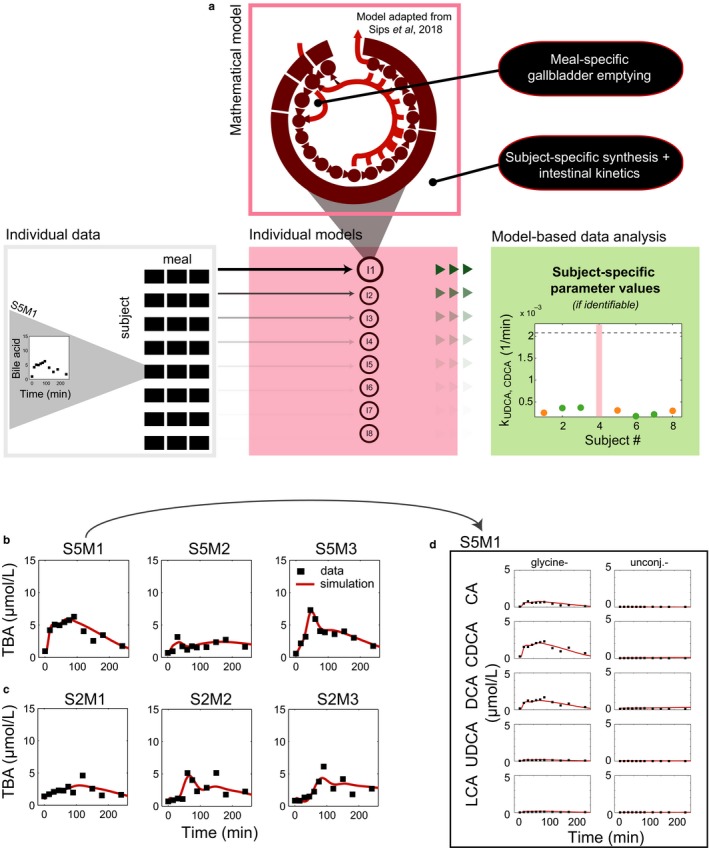
Individual total bile acid profiles and model simulation. (a) The workflow for the analysis of postprandial profiles with individual mathematical models (b) Total bile acid profiles and simulations for subject 5 and model I5. Subject 5 was selected, because the intra‐CV of the peak concentration is highest in this subject. Data are visualized with black squares, model simulation is shown as a red line. (c) As (b), for subject 2. Subject 2 had the lowest intra‐CV of the peak concentration. (d) Individual bile acid profiles underlying the total bile acid profiles for the first meal administered to subject 5

Next, with individual bile acid concentrations from the three MMTs, subject‐specific models were generated from the mathematical model of Sips et al. for further data analysis (Figure 3 A). The subject‐specific models described fasting and heterogeneous postprandial bile acids well (Figure 3b–d). Both the *interindividual* variability and the *intraindividual* variability are reproduced in model simulations. Notably, the models continue to perform well when *intraindividual *variability in the size and shape of the peak response is high (e.g., when the intra‐CV of the peak concentration is at its highest, Figure 3b). The employed differences between meal simulations—the allocation of meal specific gallbladder kinetic parameters—is thus sufficient to allow replication of *intraindividual* differences in the postprandial response.

### 3.6 Model‐based analysis of interindividual variability

**Figure 4:**
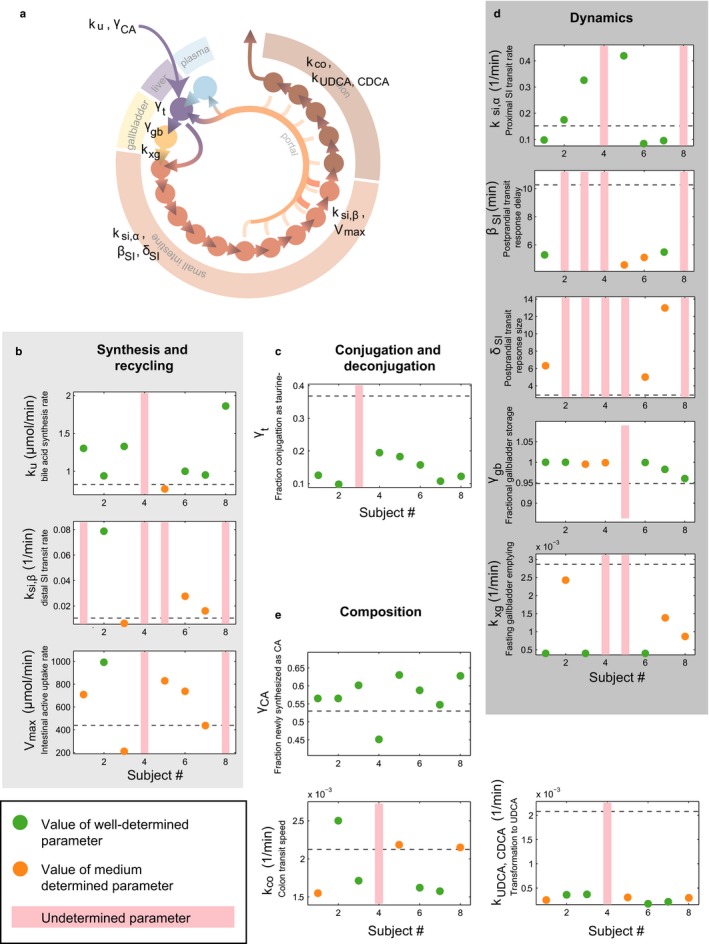
Characterization of individual bile acid metabolism via individual models. (a) Overview of the mathematical model and parameters (as presented in (Sips et al., 2018), see also Appendix 1). (b) Identifiability and values of parameters responsible for synthesis and recycling of bile acids. Green markers represent well‐identified parameters, orange markers represent parameters that have a larger range, and red bars indicate unidentifiability. The dashed line indicates the parameter value found for population level bile acid dynamics in (Sips et al., 2018). (c) As (b), for parameters governing in conjugation and deconjugation. (d) As (b), for parameters determining the (postprandial) dynamics of the bile acid pool. (e) As (b), for parameters involved in composition

As the *intraindividual* variability is sufficiently explained by meal‐specific parameters, we next examined the subject specific parameters to investigate *interindividual variability* (Figure 4). Before analysis of parameter values, we evaluated parameter identifiability to exclude unidentifiable parameters. Parameters that governed bile acid synthesis rate, composition, and intestinal transit speed were generally well determined and underlie variability between personal models.

To better understand how model parameters may reflect *interindividual* differences in bile acid metabolism, we examined the individual models for subject 3 (I3) and subject 4 (I4). These subjects are both noticeable for a large CV in fasting bile acid levels (Table S1). Furthermore, the postprandial responses of subject 3 were qualitatively distinct, as they were extended and elevated.

**Figure 5:**
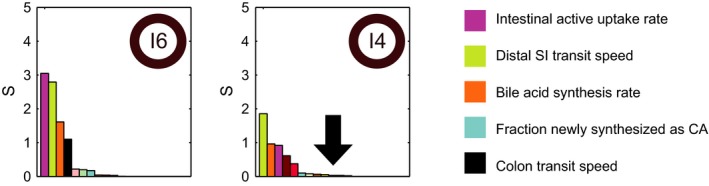
Subject 4 is distinguished by insensitivity to colon transit speed. Ranked local sensitivity of the parameters for the individual models for a typical subject (I6) (left) and I4 (right). For all subjects, the three most sensitive parameters are the bile acid synthesis rate, the intestinal active uptake rate and the distal small intestinal transit speed (the order differs between subjects, data not shown). For all models apart from I4, the fourth most sensitive parameter is the colon transit speed, which is shown in black. For I4, however, the sensitivity of the colon transit speed is found to have less influence on the simulation, indicating that for this subject only, colon transit is not a determining factor. Note that the arrow denotes the location of the black bar (colon transit speed) for I4

Analyses of individual parameter values suggested a combination of slow gallbladder emptying, slow distal small intestinal transit, and low capacity for active uptake from the intestines underlies the characteristic postprandial response of subject 3 (Figure S1). Conversely, the personal model for subject 4 (I4) could be distinguished easily from the other models by model identifiability. The majority of I4’s parameters could not be uniquely determined (Figure 4). Synthesis was also nontypical in composition, as the ratio of synthesized CA to CDCA in I4 was found to be low relative to the other subjects’ models (Figure 4). To further illustrate this qualitative difference between the personal model for subject 4 and the models of the remaining subjects, we performed local sensitivity analyses with each subject specific model (Figure 5). The ranked sensitivities for most individual models were similar, indicating that although quantitative differences between subjects may underlie *interindividual* variability, bile acid metabolism was qualitatively similar between models. The colon transit parameter, however, which held a consistently high rank in most models, was found to be of little importance in the personal model for subject 4.

## 4 DISCUSSION

In this study, we quantified *inter*‐ and *intraindividual* variability of the postprandial bile acid response in plasma of eight healthy lean men and found considerable *inter*‐ and *intraindividual* variability. This variation masked statistically significant differences between test days. Deeper, personalized, mathematical modeling analysis explained the *inter*‐ and *intraindividual* variability and provided a wealth of information on individual responses that were lost when all data were pooled. To reproduce postprandial *intraindividual* differences with the personal models, it was sufficient to allow *intraindividual* variation in gallbladder emptying parameters. Quantitative and qualitative differences in intestinal bile acid metabolism were found to underlie *interindividual* variability which was not incorporated in the gallbladder emptying parameters.

Previous studies have repeatedly described variability of postprandial plasma levels of different enteroendocrine factors such as glucose and insulin (Hall et al., 2018; Zeevi et al., 2015). Interestingly, these studies have highlighted an association between high postprandial glycemic variability and cardiometabolic risk (Hall et al., 2018; Jung, 2015), emphasizing the relevance of postprandial variability for metabolic health and thus the need for better quantification and analysis of postprandial variability (Rozendaal et al., 2018). High *interindividual* variability of postprandial bile concentrations is reported in the literature (Al‐Khaifi et al., 2018; Eggink et al., 2017; Fiamoncini et al., 2017; Sonne et al., 2016; Steiner et al., 2011). Sonne et al. investigated the postprandial response to four different liquid meals in patients with T2DM and controls and showed the wide variety of bile acid dynamics (Sonne et al., 2016). This *interindividual variability* can result from several individual factors (e.g., circadian rhythm, microbiome, diet, meal composition) (Shapiro, Kolodziejczyk, Halstuch, & Elinav, 2018; Sonne et al., 2016). In contrast, *intraindividual* variability of postprandial bile acid metabolism has hardly been investigated. In 1978, La Russo et al. reported that the postprandial plasma levels of cholyl‐conjugated cholic acid during three meals were qualitatively, but not quantitatively, similar in each subject (*N* = 4) (LaRusso, Hoffman, Korman, Hofmann, & Cowen, 1978). More recently, several aspects of the *intraindividual* variability of bile acid profiles have been examined. Steiner et al. reported high *intraindividual* variability of individual bile acids in four healthy volunteers over a 24‐hr time period (Steiner et al., 2011). However, none of these studies tried to explain the *intraindividual* variability of the postprandial response itself.

The postprandial response of unconjugated bile acids showed high *inter‐* and *intraindividual* variability that followed a different pattern compared with the conjugated bile acids. This can be explained by the fact that the unconjugated bile acids follow a diurnal rhythm which relies on colonic activity and changes in the microbiome, independently of nutrient ingestion (Al‐Khaifi et al., 2018; Steiner et al., 2011).

The unique characterization of the *intraindividual* variability allowed us to develop an individual mathematical modeling procedure for further data analysis, based on the population‐level model we have developed previously (Sips et al., 2018). To describe postprandial *intraindividual* variability with individual models, we divided the parameters into subject‐specific and meal‐specific parameters. We then hypothesized that *intraindividual* variability in gallbladder kinetics would be sufficient to describe the *intraindividual variability* in the postprandial bile acid response, because both stomach (Yokrattanasak et al., 2016) and gallbladder (Schiedermaier et al., 1997) emptying display high *intraindividual* variability. Since gallbladder emptying is a major contributor to the postprandial response, the variability herein is a natural source of postprandial variability (Sips et al., 2018). The implemented meal‐specific gallbladder kinetics were indeed sufficient to explain the observed heterogeneity of postprandial responses. In describing the *intraindividual* postprandial variability with such mechanistic details, we take a fundamentally different approach to the purely data‐driven analysis of the variability described by Hall et al. (Hall et al., 2018).

Several models of bile acid metabolism have been developed previously including the compartmental models developed by Hofmann et al. (Hofmann, Molino, Milanese, & Belforte, 1983; 1987; Molino, Hofmann, Cravetto, Belforte, & Bona, 1986; Cravetto, Molino, Hofmann, Belforte, & Bona, 1988), the more recent exogenous UDCA (Zuo, Dobbins, O'Connor‐Semmes, & Young, 2016) and endogenous LCA (Woodhead et al., 2014) focused models, and an individual, data‐driven model of postprandial total bile acid responses (Guiastrennec et al., 2018). The latter is the most similar to the approach presented here. However, in contrast to the use of total bile acid responses in (Guiastrennec et al., 2018), we included the major individual bile acids and their conjugates. In vivo*,* differences in bile acid hydrophobicity translate into differences in intestinal and hepatic uptake kinetics such as higher passive uptake of unconjugated bile acids from the intestinal lumen (Krag & Phillips, 1974) and the well‐characterized differences in hepatic extraction rates (Gilmore & Thompson, 1981; Marigold, Bull, Gilmore, Coltart, & Thompson, 1982). Therefore, postprandial dynamics and distribution differ between individual bile acids and this is relevant for bile acid receptor signaling. The use of the individual bile acids allowed us to incorporate these differences into the model and increase confidence in the calculated model parameters.

In the models, the synthesis rate, small intestinal active bile acid uptake, and colonic transit and uptake appear as the main determinants of the bile acid pool in most subjects. The models also suggest that quantitative and qualitative differences between subjects are sources for the *interindividual* variability of (postprandial) plasma bile acid levels. For example, subject 4’s postprandial response, which has a high proportion of primary bile acids, cause widespread uncertainty in the parameters of this individual's model. The individual model suggests that the absence of a colonic component in the bile acid circulation, that is, the absence of any meaningful microbial conversion or the absence of passive uptake from the colon, explains this high proportion of primary bile acids. Similarly, in subject 3 distal small intestinal active uptake is slow in the personal model, which may indicate delayed ASBT mediated bile acid uptake. If these assumptions are true, qualitative differences can be identified from postprandial plasma profiles. This enables a personalized approach because models then predict how interventions targeted at the components of our model affect the postprandial bile acid response.

Our study has some limitations. First, we only included a limited number of healthy lean males. Additionally, postprandial bile acid responses may be different in women (Fiamoncini et al., 2017). Variability in the data was assessed using coefficient of variance (CV) of the AUCs and iAUCs, individual time points and peak concentrations. Other data‐driven metrics for time‐series analysis could have been considered. However, methods such a spectral clustering, or mixed‐effect models require more data (more individuals and more time samples) (Berglund, Adiels, Taskinen, Borén, & Wennberg, 2015; Hall et al., 2018). We used a liquid mixed meal in our study because of practical and standardization purposes, but the postprandial response (i.e., intestinal motility and hormonal response) to solid food is slightly different compared to a liquid meal (Camilleri, 2006).

Furthermore, our modeling analysis is based on only peripheral plasma samples and does not include measurements on bile or feces, nor portal vein samples. This limits the validation of the personalized models in this study. We previously calibrated and validated the model with a wide selection of (nonplasma) data (Sips et al., 2018), however, the model underwent several minor adaptations (Appendix 1) and was subsequently identified with plasma bile acid data only. This approach reproduced the variation in the dataset, produced consistent results for different simulation approaches and was carefully evaluated for identifiability and consistency. Nevertheless, additional validation data (e.g., gallbladder emptying or fecal bile acids) are preferred in the future.

Finally, the modeling approach distinguishes different contributions to postprandial variability, however, the *intraindividual* variability of fasted bile acids concentrations is not yet incorporated. Factors underlying *intraindividual* variability of fasting bile acid metabolism include diet (DenBesten, Connor, & Bell, 1973; Nilsson, Östman, Holst, & Björck, 2008) and sleep quality (Ferrell & Chiang, 2015; Morgan, Hampton, Gibbs, & Arendt, 2003). It is possible to incorporate these factors in the models for future studies.

## 5 CONCLUSIONS

In summary, this study was performed to assess the variability of the bile acid response to mixed meal testing in healthy men. The data presented here characterize *the inter*‐ and *intraindividual* variability of the postprandial bile acid response. More so, the mathematical models allocated the *interindividual* variability to distal active uptake, colon transit, and microbial bile acid transformation, whereas for *intraindividual* variability, it was sufficient to allow variation in gallbladder kinetics. Personalized mathematical modeling may thus allow us to identify qualitative and quantitative characteristics of individual bile acid metabolism based on postprandial plasma bile acid responses alone.

## CONFLICT OF INTERESTS

None.

## AUTHORS CONTRIBUTIONS

ECEM recruited the subjects, performed the clinical experiments, did statistical analysis, wrote and edited the manuscript. FLPS did statistical analysis and applied the mathematical modeling, wrote and edited the manuscript. HME performed clinical experiments and reviewed the manuscript. MK performed laboratory analysis of data and reviewed the manuscript. JAR reviewed the manuscript. AKG and NAWR reviewed and edited the manuscript. MRS designed the study, reviewed and edited the manuscript.

## APPENDIX 1

### Gallbladder emptying function

The gallbladder emptying function presented in (Sips et al., 2018) was designed to represent the response to a nonspecified large meal, averaged over the healthy population. To facilitate modeling of the (nonuniform) response to the liquid meal at an individual level, we implemented an extension of the gallbladder emptying function in (Sips et al., 2018), so that the flux $r_{si1,gb}^{\mathrm{BA}}$ of bile acid BA from the gallbladder gb to duodenal compartment $si_{1}$ is now described by Equation 1.

(1) $$r_{si1,gb}^{\mathrm{BA}}=k_{\mathrm{xg}}\cdot \rho_{2}\left(\left(t-\tau \right),\beta_{gb1},\delta_{gb1},\beta_{gb2},\delta_{gb2}\right)\cdot BA_{\mathrm{gb}}.$$

Herein, $k_{\mathrm{xg}}$(1/min) is the gallbladder emptying rate in the fasting state, $BA_{\mathrm{gb}}$ is the gallbladder content of BA (µmol), τ is the time of the meal, and $\rho_{2}\left(t,\beta_{1},\delta_{1},\beta_{2},\delta_{2}\right)$ is a normalized double Rayleigh function which shows a transient, nonuniform increase, characterized by peak locations β (min) and relative increases of $\delta \left(\rho_{2}\left(t,\beta_{1},\delta_{1},\beta_{2},\delta_{2}\right)=1+\delta_{1}\cdot \left(\frac{t\sqrt{e}}{\beta_{1}}e^{-\frac{t^{2}}{2\cdot \beta_{1}^{2}}}\right)+\delta_{2}\cdot \left(\frac{t\sqrt{e}}{\beta_{2}}e^{-\frac{t^{2}}{2\cdot \beta_{2}^{2}}}\right)\right)$. When $\delta_{gb2}$ is set to 0, the original form of the gallbladder emptying function is obtained.

To simulate the model, $\beta_{gb1},\delta_{gb1},\beta_{gb2},\;\text{and}\;\delta_{gb2}$ are defined as the *meal*‐specific parameters, whereas all other parameters are subject specific. To initiate the model with all bile acid species at their basal, fasting levels, the MMT simulations that are presented are preceded by an equilibration simulation in which identical meals are given three times a day until subsequent responses are identical, as described in (Sips et al., 2018). In total, 14 gallbladder emptying parameters are estimated from the data—the four gallbladder emptying parameters for each of the three MMT *and* $\beta_{gb1}\;\text{and}\;\delta_{gb1}$ as used to simulate the model in the equilibration period (for these unobserved meals, $\delta_{gb2}$ is set to 0).

### Model adaptations

Further adaptations of the model are:

1. Since sulfated LCA was not determined and the sulfation pathway plays a minor role in healthy bile acid metabolism, sulfated LCA ($LCA_{s}$) and the (de‐) sulfation parameters $k_{LCA,LCA_{s}}$ and $k_{LCA_{s},LCA}$ were removed from the model.
2. The following parameters were fixed to the values determined in (Sips et al., 2018)
  - Liver output parameter $k_{\mathrm{xl}}$—because liver output is fast and plasma measurements are not sensitive to its value.
  - Colon passive uptake parameter $k_{xi,up,co}$—we assume that passive diffusion across the colonocytes is constant, hereby bounding the colon model to physiological values and preventing unidentifiability of fecal output.
  - All hepatic extraction parameters—as they were determined accurately and validated in (Sips et al., 2018), and show little variation over a healthy population (Eggink et al., 2017).

### Optimization and sensitivity

To estimate the free parameters of the model, the cost function was based on the squared difference between the simulated (model output y for subject s, MMT m, time point tp, bile acid species ba and parameter vector p); $y_{s,m,tp,ba}\left(p\right)$ and measured individual bile acid concentrations (datapoint $d_{s,m,tp,ba}$).

(2) $$E_{s}\left(p\right)=\sum_{m=1}^{M}\sum_{tp=1}^{\mathrm{TP}}\sum_{ba=1}^{\mathrm{BA}}{\left(y_{s,m,tp,ba}\left(p\right)-d_{s,m,tp,ba}\right)}^{2}$$

The cost function further includes penalties that serve to regularize the model's behavior in the states for which no data are available and the equilibration period.

(3) $$CF_{s}\left(p\right)=E_{s}\left(p\right)+\sum_{n=1}^{\mathrm{nP}}p_{n,s}\left(p\right)$$

In total, we incorporate two regularization penalties (*nP* = 2)—the first to prevent accumulation of unobserved other bile acid species o to more than 25% (i.e., $thr_{o}=0.25)$ of the sum of CA, CDCA, DCA, UDCA and o itself as calculated in the fasting state ($t_{f})$:

(4) $$p_{1}=\left\{\begin{array}{cc} \frac{o\left(t_{f},\;p\right)}{\sum BA\left(t_{f},\;p\right)}\cdot 1e12 & if\frac{o\left(t_{f},\;p\right)}{\sum BA\left(t_{f},\;p\right)}>thr_{o}\\ 0 & if\frac{o\left(t_{f},\;p\right)}{\sum BA\left(t_{f},\;p\right)}\leq thr_{o} \end{array}\right..$$

The second to penalize insufficient gallbladder emptying in an equilibration period day by means of a threshold $thr_{e}$, which is set to 65% (Sorenson, Fancher, Lang, Edit, & Ralph Broadwater, 1993) and is compared to the minimal gallbladder content ($GB\left(t\right))$ relative to the fasting content $GB_{f}$.

(5) $$p_{2}=\left\{\begin{array}{cc} \frac{\min\;\left(GB\left(t\right)\right)}{GB_{f}}\cdot 1e12 & if\frac{\min\;\left(GB\left(t\right)\right)}{GB_{f}}\cdot 100\%>thr_{e}\\ 0 & if\frac{\min\;\left(GB\left(t\right)\right)}{GB_{f}}\cdot 100\%\leq thr_{e} \end{array}\right..$$

The model was implemented in Matlab (2012a/2016a, The MathWorks, Natick, Massachusetts). The differential equations were solved using MEX‐files compiled with the aid of the SUNDIALS CVode package (2.6.0, Lawrence Livermore National Laboratory, Livermore, California) (Hindmarsh et al., 2005). For parameter estimation, nonlinear least squares optimizer LSQNONLIN was used.

A local sensitivity parameter measure $S_{s,p_{k}}$ of parameter $p_{k}$ for subject s was determined via

(6) $$S_{s,p_{k}}=\frac{1}{2\Delta }\left({\left(STBA_{s}\left(p_{k}\right)-STBA_{s}\left(p_{k}+\Delta p_{k}\right)\right)}^{2}+{\left(STBA_{s}\left(p_{k}\right)-STBA_{s}\left(p_{k}+\Delta p_{k}\right)\right)}^{2}\right)$$

where $STBA_{s}\left(p\right)=\sum_{m=1}^{M}\sum_{tp=1}^{\mathrm{TP}}\sum_{ba=1}^{\mathrm{BA}}y_{s,m,tp,ba}\left(p\right).$
