# Supplementary figures and images for: Model‐based data analysis of individual human postprandial plasma bile acid responses indicates a major role for the gallbladder and intestine
Source: Physiol Rep. 2020 Mar 13;8(5):e14358. doi: 10.14814/phy2.14358 (PMC7070101; doi:10.14814/phy2.14358)

**A**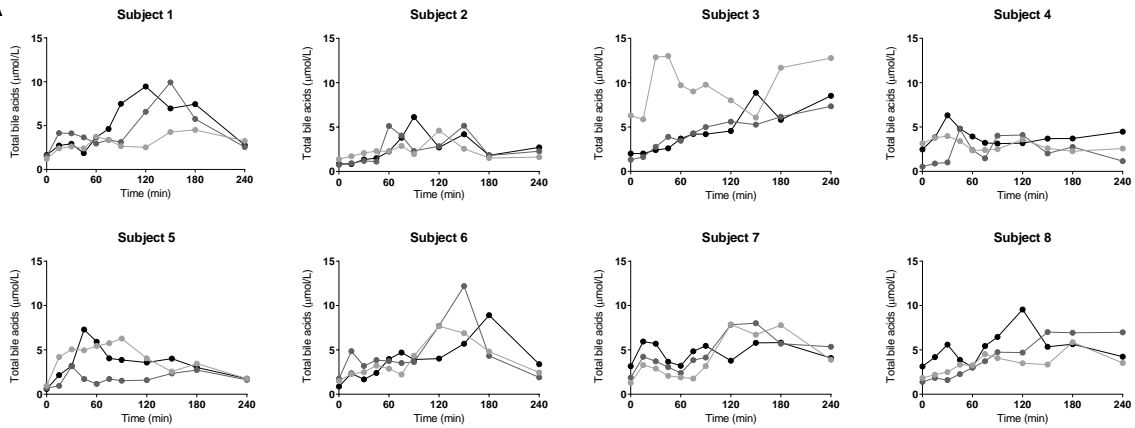**B**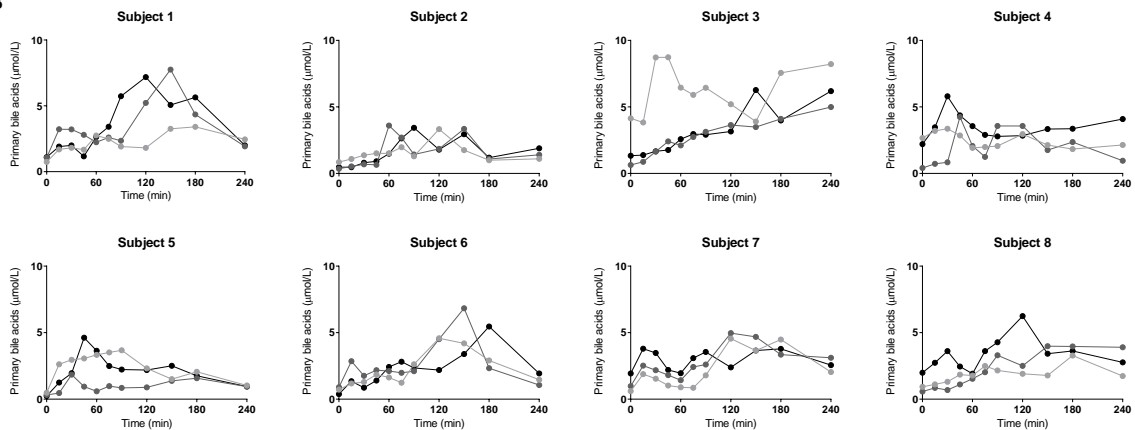**C**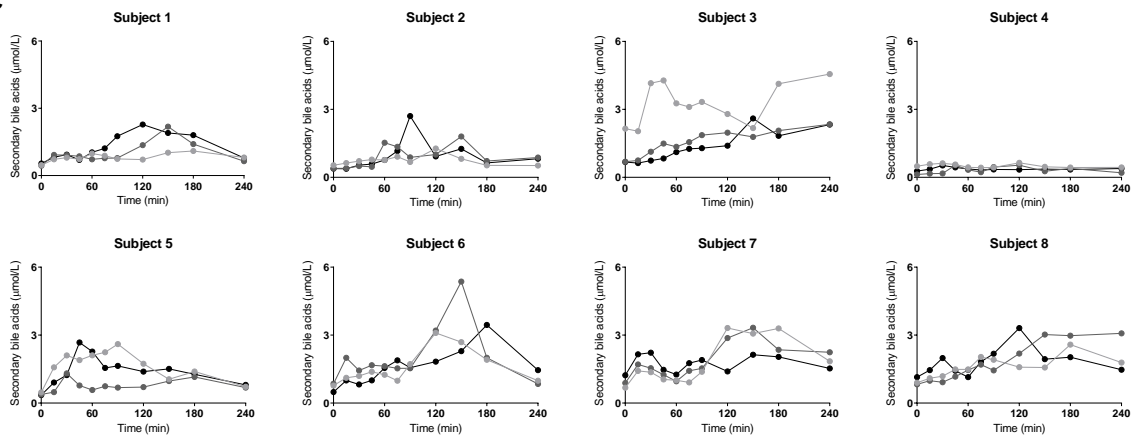

**D**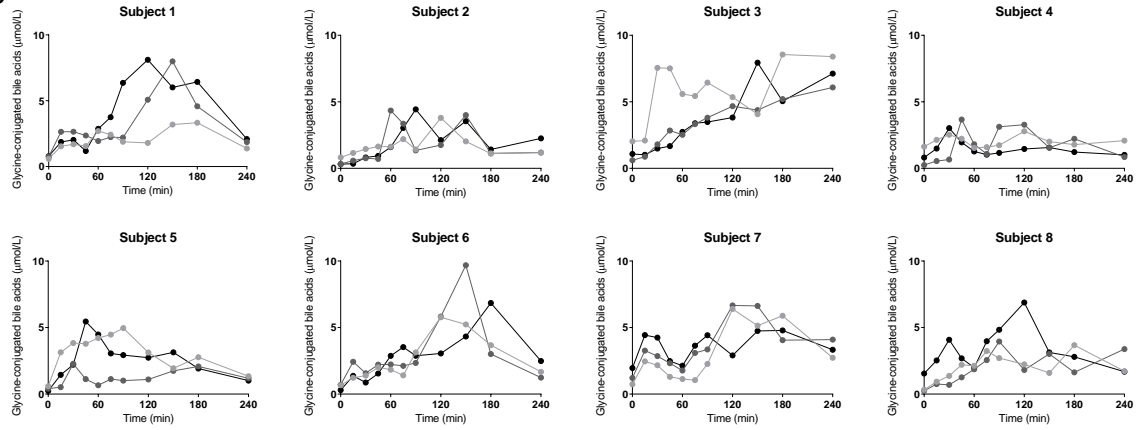**E**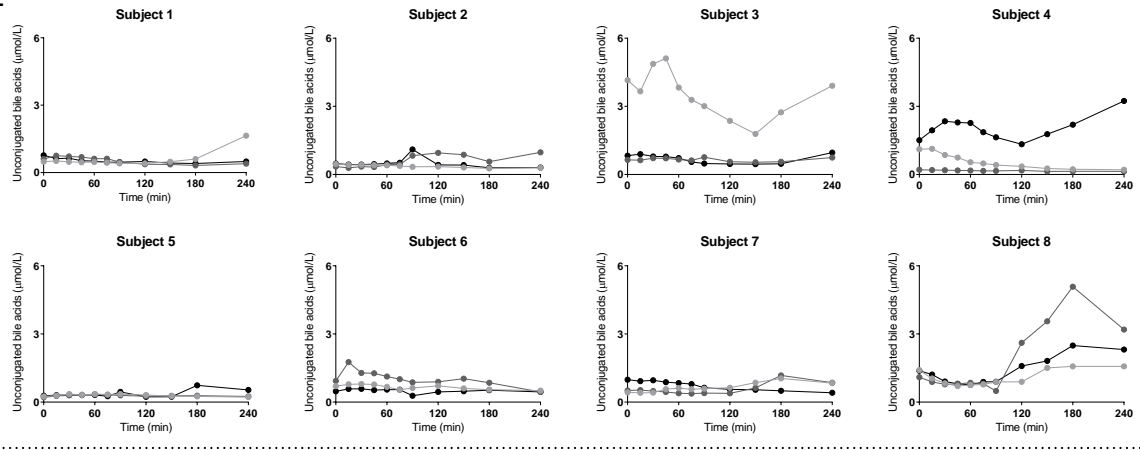

Supplement: Supplementary file 1 [file PHY2-8-e14358-s001.pdf]
